# Аденомы гипофиза: путь к пониманию агрессивной формы. Клинико-генетический анализ потенциальных прогностических маркеров в развитии агрессивных аденом гипофиза

**DOI:** 10.14341/probl13487

**Published:** 2025-09-14

**Authors:** З. Ю. Халимова, О. Т. Азимова

**Affiliations:** Республиканский специализированный научно-практический медицинский центр эндокринологииим. акад. Ё.Х. Туракулова; Republican Specialized Scientific and Practical Medical Center of Endocrinology named after Acad. Y. Kh. Turakulov

**Keywords:** аденомы гипофиза, агрессивные аденомы гипофиза, генетические маркеры, ген VEGFA, ген TP53_2, ген HIF1A, ген IL-17A, pituitary adenomas, aggressive pituitary adenomas, genetic markers, VEGFA gene, TP53_2 gene, HIF1A gene, IL-17A gene

## Abstract

**BACKGROUND:**

BACKGROUND. Currently, due to the lack of clear criteria for predicting the aggressive course of pituitary adenomas (APA), the search for diagnostic markers is highly relevant. Genetic markers, among others, may serve as such markers since their identification is possible at early stages of the pathological process.

**OBJECTIVE:**

OBJECTIVE. To study the prevalence of genotypic polymorphisms G634C of the VEGFA gene (locus rs2010963), C/T of the TP53_2 gene (locus rs17884159), C/T of the HIF1A gene (locus rs11549465), and G-197A of the IL-17A gene in a sample of patients with APA and their association with the development of various clinical variants of the aggressive course of the disease.

**MATERIALS AND METHODS:**

MATERIALS AND METHODS. The study included 100 patients with a clinically confirmed diagnosis of pituitary adenoma (main group) and 83 practically healthy individuals (control group). The polymorphism of the studied genes was analyzed using allele-specific polymerase chain reaction (PCR) with SNP-Express reagent kits in real-time mode ("Sintol", Russia). The interpretation of the results was carried out using the "RotorGene" software of the PCR-RV device. The study also included general clinical, biochemical, and hormonal tests, as well as instrumental and neuroimaging methods, including magnetic resonance imaging (MRI) of the chiasmatic-sellar region and statistical analysis.

**RESULTS:**

RESULTS. The study showed that the heterozygous mutation (G/C) of the G634C VEGFA polymorphism was recorded in 21 cases (26%), and the homozygous mutation with a complete replacement of guanine (G) by cysteine (C) at position 634 (C/C) was detected in 4 cases. In patients with invasive pituitary adenomas (PA), the heterozygous variant (G/C) was twice as frequent — 32.7% (n=17) compared to the control group — 15.7% (n=13). The homozygous genotype (C/C) was also more frequently observed in patients with invasive PA growth — 7.7% (n=4) compared to the control group.

The heterozygous variant (C/T) of the HIF1A gene was significantly more common (p=0.02) in patients with invasive adenomas compared to the control group: 25% (n=13) and 9.8% (n=8), respectively. In non-invasive PAs, this genotype was observed three times less frequently. The study of TP53_2 polymorphism (locus rs17884159) showed that in patients with invasive PAs, the frequency of the heterozygous variant (C/T) was significantly higher — 15.4% (n=8) compared to the control group — 4.8% (n=4).

**CONCLUSION:**

CONCLUSION. The conducted genetic analysis of polymorphisms in the VEGFA, HIF1A, TP53_2, and IL-17A genes revealed significant deviations, confirming their practical significance in the early diagnosis of aggressive pituitary adenomas.

## ОБОСНОВАНИЕ

Аденомы гипофиза — это часто встречающиеся новообразования, большинство из которых имеют доброкачественную природу и остаются клинически незначимыми. Клинически значимые аденомы развиваются в результате соматических и герминативных мутаций, характеризуются неконтролируемой секрецией гормонов и ростом самой опухоли [1][2]. В некоторых случаях аденомы могут проявлять агрессивное течение, однако их злокачественное перерождение встречается крайне редко. Гормонально-активные аденомы клинически проявляются в виде акромегалии, гигантизма, болезни Кушинга и других патологических состояний, характеризующихся избыточной секрецией гормонов [3].

Термин «агрессивная аденома гипофиза» (ААГ) имеет разные трактовки и применяется для обозначения АГ, характеризующихся инвазивным ростом, крупными размерами (гигантские аденомы) или резистентностью к специфической терапии. Однако консенсус по данному термину до сих пор не достигнут, что затрудняет объективную оценку реальной эпидемиологической ситуации [4][5]. Хотя агрессивные аденомы гипофиза чаще всего диагностируются как макроаденомы, размер опухоли не всегда коррелирует с агрессивным течением, как это демонстрируют гигантские лактотропные опухоли, которые могут быть высокочувствительными к медикаментозному лечению. Более того, эффективность хирургического вмешательства не зависит исключительно от размеров опухоли [6][7][8].

Распространенность ААГ оценивалась на основе анализа серий клинических случаев. Согласно имеющимся данным, доля инвазивных АГ и послеоперационных рецидивов составляет около 2% среди макроаденом, причем эта пропорция выше для секреторных опухолей [4]. В настоящее время в Узбекистане создан единый национальный регистр аденом гипофиза (АГ), включающий статистические данные всех пациентов, обратившихся в Республиканский специализированный научно-практический медицинский центр эндокринологии им. акад. Ё.Х. Туракулова (Свидетельство о регистрации базы данных в агентстве по интеллектуальной собственности РУЗ № BGU 00399). По данным регистра, агрессивные формы АГ составляют от 25 до 50% всех случаев и требуют особого внимания.

В настоящее время клинические исследования уделяют повышенное внимание критериям агрессивного течения АГ, что нашло отражение в 5-й редакции классификации Всемирной организации здравоохранения (ВОЗ) от 2022 г. [2][7]. Не существует единого мнения относительно критериев быстрого роста АГ, который является одним из признаков агрессивного течения. Подобно другим солидным опухолям, для объективной оценки размеров аденом гипофиза может применяться методология RECIST 1.1, позволяющая точно оценивать ответ опухоли на терапию, так как этот показатель коррелирует с объемом опухоли. Согласно этим критериям, значительным ростом опухоли считается увеличение диаметра на 20%. Однако рост АГ можно считать аномальным только в случаях, когда значительное увеличение размеров происходит в течение определенного периода [6][9].

Главным ограничением в оценке агрессивного течения АГ является отсутствие прогностических маркеров с высокой степенью чувствительности [10][11][12]. Согласно одной из классификаций ВОЗ, критерием агрессивности и инвазивности опухоли является показатель пролиферации, который определяется по числу митозов и индексу Ki-67. Однако пороговые значения для этих параметров пока не установлены. Несмотря на то, что наличие данных признаков коррелирует с высоким риском и неблагоприятным прогнозом, данный показатель не всегда адекватно отражает особенности клинического течения АГ. Позже, в дополнение к гистологической градации ВОЗ, была предложена пятиуровневая система прогнозирования клинического течения АГ, включающая показатели инвазии и пролиферации, такие как митотический индекс >2, Ki-67 ≥3% и иммунопозитивность p53. Эта система оценки была проверена в четырех независимых когортных исследованиях, охвативших 1992 пациента. Опухоли класса 2b (инвазивные и пролиферативные), составляющие от 5,4 до 8,8% хирургических серий, ассоциируются с повышенным риском рецидива или прогрессирования, несмотря на медикаментозную терапию [6][13][14].

В связи с вышеизложенным, поиск специальных критериев для прогнозирования агрессивного течения АГ является актуальным и востребованным направлением научных исследований, имеющим важное практическое значение.

## ЦЕЛЬ ИССЛЕДОВАНИЯ

Изучение распространенности генотипических полиморфизмов G634C гена VEGFA (локус rs2010963), C/T гена TP53_2 (локус rs17884159), C/T гена HIF1A (локус rs11549465) и G-197A гена IL-17A в выборке пациентов с агрессивными аденомами гипофиза (ААГ), а также их связи с развитием различных клинических вариантов агрессивного течения заболевания.

## МАТЕРИАЛ И МЕТОДЫ ИССЛЕДОВАНИЯ

Работа проводилась на базе Республиканского специализированного научно-практического медицинского центра эндокринологии им. академика Ё.Х. Туракулова МЗ.РУз. Исследуемая выборка пациентов с АГ была отобрана из созданного на базе центра регистра пациентов с АГ.

## Характеристика клинического материала

Критерии включения (основная группа): гормонально активные и гормонально неактивные аденомы с эндо-, инфра-, супраселлярным и тотальным ростом, микроаденомы (размером менее 1 см) и макроаденомы (размером более 1 см) и гигантские аденомы (размером более 4 см), имеющие агрессивное течение (рецидивирующее, резистентность, быстрый рост и визуализационных характеристик агрессивного роста).

Критерии исключения: онкологический анамнез, сопутствующие генетические заболевания.

По результатам отбора в основную группу было включено 52 больных с ААГ.

29 пациентов с АГ без признаков агрессивного течения были включены в группу сравнения.

В качестве контрольной группы была отобрана однородная по полу и возрасту группа из 83 субъектов, у которых нет клинических признаков АГ.

Возрастная и гендерная характеристики пациентов в исследуемых группах представлены в таблице 1.

**Table:** Таблица 1. Возрастная и гендерная характеристики пациентов в исследуемых группах

	Основная группа — пациенты с ААГ (n=52)	Сравнительная группа —пациенты с АГ без признаков агрессивного течения (n=29)	Контрольная группа — здоровые лица (n=83)
%	n	%	n	%	n
Мужчины	34	65,3	17	58,6	53	63,8
Женщины	18	34,7	12	41,4	30	36,1
Возраст (M±m)	33,9±10,3	37,3±9,3	39,4±14,3

Основная группа, 52 больных с ААГ, была разделена на 2 подгруппы в зависимости от степени инвазии: основная группа 1 — 22 больных с инвазивными аденомами гипофиза с 1–2 степенью роста и основная группа 2–30 больных с 3–4 степенью инвазивности.

На графике 1 представлено распределение пациентов по гормональной активности опухоли.

**Figure:**
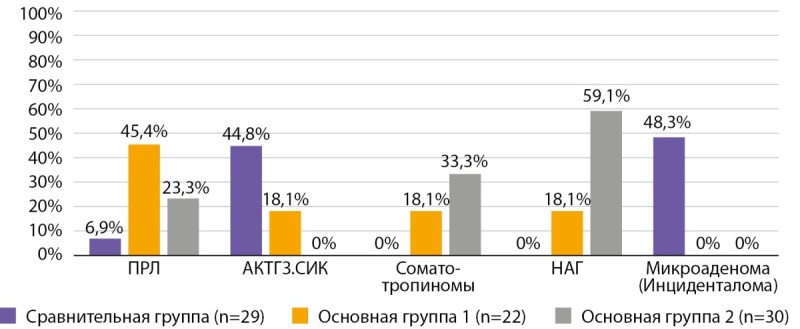
Рисунок 1. Распределение в исследуемых группах АГ по гормональной активности.

## Дизайн исследования

Исследование носило ретроспективный сравнительный характер с определением частоты встречаемости генотипических полиморфизмов G634C гена VEGFA (локус rs2010963), C/T гена TP53_2 (локус rs17884159), C/T гена HIF1A (локус rs11549465) и G-197A гена IL-17A в группах пациентов с ААГ, АГ без признаков агрессивного течения, а также здоровых лиц с оценкой связи между носительством определенного генотипа с особенностями клинического течения заболевания.

## Методы исследования

Для решения исследовательских задач использованы общеклинические, биохимические и гормональные исследования, а также специальные методы, включая молекулярно-генетический анализ полиморфизмов с применением полимеразно-цепной реакции в реальном времени (ПЦР-РВ). Кроме того, применялись инструментальные и нейровизуализационные методы, такие как магнитно-резонансная томография (МРТ) хиазмально-селлярной области, а также статистические методы анализа данных.

Молекулярно-генетические исследования проводились на базе лаборатории молекулярно-генетического отдела Специализированного научно-практического медицинского центра гематологии Министерства здравоохранения Республики Узбекистан. Материалом исследования служила кровь пациентов. Определение полиморфизмов генов G634C VEGFA (локус rs2010963), C/T TP53_2 (локус rs17884159), C/T HIF1A (локус rs11549465) и G-197A IL-17A выполнялось методом аллель-специфичной полимеразной цепной реакции (ПЦР). Для исследования использовались наборы реагентов SNP-экспресс в режиме real-time («Синтол», Россия). Интерпретация полученных данных осуществлялась с использованием программного обеспечения RotorGene прибора ПЦР-РВ (RT-PCR).

## РЕЗУЛЬТАТЫ

Осуществлен сравнительный анализ частоты встречаемости генотипов полиморфизмов генов VEGFA, ТР53, HIF-1α, IL-17A в трех исследуемых группах: неивазивные АГ (29 больных), инвазивные АГ (52 больных) и контрольная группа (83 здоровых лиц) (табл. 2–5).

**Table:** Таблица 2. Частота распределения генотипов полиморфизма G634C гена VEGFA (rs2010963) в исследуемых группах

Генотип	Контрольная группа n=83 (%)	Сравнительная группа n=29 (%)	OR (95%CI)	p	Контрольная группа n=83 (%)	Основная группа n=52 (%)	OR (95%CI)	p
G/G	68 (81,9)	25 (86,2)	1,37 (0,4–4,5)	0,59	68 (81,9)	31 (59,6)	0,32 (0,14–0,71)	0,005
G/С	13 (15,7)	4(13,8)	0.86 (0,25–2,8)	0,8	13 (15,7)	17 (32,7)	2,6 (1,14–5,98)	0,02
C/C	2 (2,4)	-	-	-	2 (2,4)	4 (7,7)	3,3 (0,6–19,1)	0,17

**Table:** Таблица 3. Частота распределения генотипов полиморфизма C/T гена HIF1α (rs11549465) в исследуемых группах

Генотип	Контрольная группа n=83 (%)	Сравнительная группа n=29 (%)	OR (95%CI)	p	Контрольная группа n=83 (%)	Основная группа n=52 (%)	OR (95%CI)	p
C/C	74 (89,2)	25 (86,2)	0,76 (0,21–2,68)	0,67	74 (89,2)	36 (69,2)	0,27 (0,11–0,67)	0,005
C/T	8 (9,6)	4 (13,8)	1,5 (0,4–5,4)	0,53	8 (9,6)	13 (25,0)	3,1 (1,2–8,1)	0,02
T/T	1 (1,2)	0 (0,0)	-	-	1 (1,2)	3 (5,8)	5,0 (0,5–49,6)	0,16

**Table:** Таблица 4. Частота распределения генотипов полиморфизма C/T генаTP53_2 (rs17884159) в исследуемых группах

Генотип	Контрольная группа n=83 (%)	Сравнительная группа n=29 (%)	OR (95%CI)	p	Контрольная группа n=83 (%)	Основная группа n=52 (%)	OR (95%CI)	p
C/C	79 (95,2)	26 (89,6)	2,3 (0,47–10,8)	0,30	79 (95,2)	43 (82,7)	0,24 (0,07–0,83)	0,02
C/T	4 (4,8)	3 (10,3)	2,3 (0,47–10,8)	0,30	4 (4,8)	8 (15,4)	3,6 (1,02–12,6)	0,04
T/T	0 (0,0)	0 (0,0)	-	-	0 (0,0)	1 (1,9)	-	-

**Table:** Таблица 5. Частота распределения генотипов полиморфизма G-197A в гене IL17A в исследуемых группах

Генотип	Контрольная группа n=83 (%)	Сравнительная группа n=29 (%)	OR (95%CI)	p	Контрольная группа n=83 (%)	Основная группа n=52 (%)	OR (95%CI)	p
G/G	53 (63,9)	20 (69,0)	1,25 (0,5–3,1)	0,61	53 (63,9)	29 (55,8)	0,71 (0,35–1,44)	0,34
G/A	26 (31,3)	8 (27,6)	0,83 (0,32–2,1)	0,70	26 (31,3)	19 (36,5)	1,26 (0,60–2,6)	0,53
A/A	4 (4,8)	1 (3,4)	0,70 (0,07–6,6)	0,75	4 (4,8)	4 (7,7)	1,64 (0,39–6,8)	0,49

В ходе исследования были выявлены значимые различия в частоте встречаемости генотипов полиморфизма G634C гена VEGFA между контрольной и основной группами. Генотип G/G встречался в основной группе более чем в 2,5 раза реже по сравнению с контрольной группой. В то же время генотип G/C, напротив, встречался в основной группе в 2,6 раза чаще, что могло указывать на его связь с предрасположенностью ААГ. Частота генотипа C/C также была выше в основной группе, однако различия не достигли статистической значимости, что не позволило сделать однозначные выводы о его влиянии. При сравнении контрольной и сравнительной групп значимых различий в распределении генотипов не было выявлено, что свидетельствовало об их схожести по частоте встречаемости. Таким образом, полученные результаты указывали на возможную роль генотипа G/C в повышении риска развития ААГ.

В ходе исследования частоты распределения генотипов полиморфизма C/T гена HIF1α (rs11549465) было выявлено, что генотип C/C встречался в основной группе в 3,7 раза реже по сравнению с контрольной группой. В то же время генотип C/T встречался в основной группе в 3,1 раза чаще, что указывало на его потенциальную связь с предрасположенностью к агрессивному течению АГ. Частота генотипа T/T также была выше в основной группе, однако различия не достигли статистической значимости, что не позволило сделать окончательные выводы о его влиянии. Таким образом, результаты исследования показали, что генотип C/T мог быть ассоциирован с более агрессивным течением АГ, тогда как генотип C/C, напротив, ассоциирован с АГ без агрессивного течения или с отсутствием АГ.

В ходе исследования частоты распределения генотипов полиморфизма C/T гена TP53_2 (rs17884159) было выявлено, что генотип C/C встречался в основной группе реже, чем в контрольной, почти в 4 раза. В то же время частота генотипа C/T была в основной группе в 3,6 раза выше по сравнению с контрольной группой.

В ходе исследования частоты распределения генотипов полиморфизма G-197A в гене IL17A значимых различий между контрольной и основной группами не было выявлено. Генотип G/G встречался в основной группе несколько реже по сравнению с контрольной, однако различия не достигли статистической значимости. Генотип G/A, напротив, встречался в основной группе чаще, но также без достоверных различий. Частота генотипа A/A была несколько выше в основной группе, однако статистически значимые различия отсутствовали.

Для определения взаимосвязи особенностей течения при ААГ и генетических мутациях нами была произведена дифференцировка АГ на первично инвазивные, рецидивирующие после транссфеноидальной аденомэктомии и резистентные к медикаментозной терапии (рис. 2). Генотип G/G для всех исследованных генов чаще встречался у пациентов с первично инвазивными аденомами, что может свидетельствовать о его потенциальной связи с менее агрессивным течением заболевания. В частности, при анализе гена VEGFA данный генотип доминировал среди пациентов с первичной инвазией, тогда как в группах с рецидивирующими и резистентными формами частота его встречаемости была ниже. Аналогичная тенденция наблюдалась для гена HIF-1α, где генотип G/G был наиболее распространен среди пациентов с первичной инвазией, в то время как в группе резистентных опухолей его частота снижалась.

**Figure:**
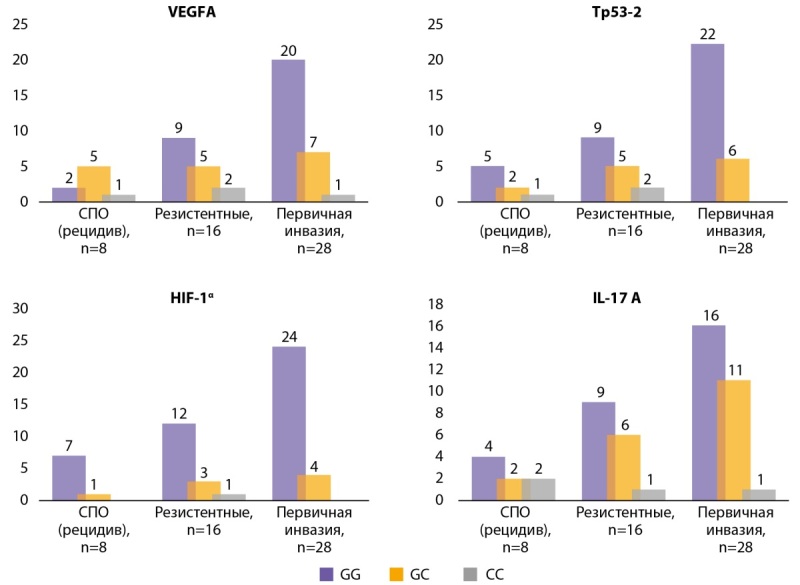
Рисунок 2. Результаты анализа взаимосвязи генетических особенностей при первично инвазивных аденомах, рецидивирующих и резистентных ААГ в сравнительном аспекте.

Генотип G/C продемонстрировал более высокую частоту среди пациентов с рецидивирующими и резистентными опухолями, особенно по генам VEGFA, TP53-2 и IL-17A, что может указывать на его возможную связь с развитием устойчивости опухоли и повышенным риском агрессивного течения. Для гена IL-17A данный генотип встречался значительно чаще в группе первично инвазивных опухолей по сравнению с рецидивами, что может свидетельствовать о его потенциальной роли в процессе опухолевой инвазии.

Генотип C/C, напротив, встречался редко во всех исследуемых группах, что может свидетельствовать о его ограниченной роли в развитии и прогрессии заболевания. Его низкая частота не позволила выявить достоверные различия между группами.

## ОБСУЖДЕНИЕ

Результаты настоящего исследования показали значительные различия в частоте встречаемости полиморфизмов генов G634C VEGFA, C/T TP53_2, C/T HIF1A и G-197A IL-17A среди пациентов с ААГ по сравнению с АГ без признаков агрессивного течения и контрольной группой. Эти данные подтверждают гипотезу о возможной генетической предрасположенности к агрессивному течению АГ и согласуются с результатами аналогичных международных исследований.

Наши результаты демонстрируют, что гетерозиготный вариант (G/C) полиморфизма G634C гена VEGFA встречается в два раза чаще у пациентов с агрессивными аденомами гипофиза (32,7%), чем в контрольной группе (15,7%). Это согласуется с данными, полученными в исследованиях Smith и соавт. (2019 г.) [15], где частота встречаемости гетерозиготных мутаций в гене VEGFA среди пациентов с инвазивными опухолями гипофиза также была выше, чем среди пациентов с доброкачественным течением. Данный факт свидетельствует о возможной роли VEGFA в ангиогенезе и прогрессии агрессивных форм АГ.

Полиморфизм C/T гена TP53_2 также продемонстрировал значимые различия между группами: частота гетерозиготного варианта была значительно выше у пациентов с инвазивными АГ (15,4%), чем в контрольной группе (4,8%). Этот результат соответствует выводам исследования Gonzalez и соавт. (2021 г.) [16], где полиморфизм гена TP53 был идентифицирован как маркер агрессивности солидных опухолей, включая опухоли гипофиза.

Известно, что белок HIF1A является ключевым регулятором гипоксического ответа опухолевых клеток, что подтверждает его участие в опухолевой инвазии. В нашем исследовании гетерозиготный вариант (C/T) гена HIF1A встречался значительно чаще среди пациентов с агрессивными АГ (25%) по сравнению с контрольной группой (9,8%). Эти данные согласуются с исследованиями Zhang и соавт. (2020 г.) [17], где частота данного полиморфизма была связана с повышенным риском агрессивного течения опухолей головного мозга.

Интересным наблюдением стало отсутствие значимых различий в распространенности полиморфизма G-197A гена IL-17A между группами пациентов с АГ и контрольной группой. Однако более высокие показатели носительства гетерозиготного варианта (G/A) среди пациентов с резистентными формами АГ (36,5%) могут указывать на потенциальную роль воспалительного процесса в развитии устойчивости к медикаментозной терапии. Этот вывод подтверждается данными, представленными в исследовании Lee и соавт. (2021 г.) [18], где повышенная экспрессия IL-17A коррелировала с резистентностью опухолевых клеток к лечению.

В настоящем исследовании подтверждена ассоциация полиморфизмов генов VEGFA, TP53_2, и HIF1A с различными клиническими вариантами агрессивного течения АГ. Пациенты с инвазивными опухолями имели более высокую частоту мутаций в генах VEGFA и HIF1A, что свидетельствует о возможном вовлечении этих генов в процессы ангиогенеза и гипоксической адаптации опухолевых клеток. В то же время полиморфизм гена TP53_2 был более характерен для пациентов с рецидивами, что подтверждает его роль в нарушении механизмов апоптоза и повышении риска рецидивирования опухоли.

Несмотря на полученные значимые результаты, настоящее исследование имеет ряд ограничений. Во-первых, относительно небольшая выборка пациентов может ограничивать обобщаемость полученных данных. Во-вторых, анализ был проведен только на четырех генетических полиморфизмах, в то время как известно, что агрессивное течение АГ может определяться множеством генетических и эпигенетических факторов.

Выявление генетических предикторов агрессивного течения АГ может способствовать персонализированному подходу к лечению пациентов. Например, пациенты с выявленными мутациями в генах VEGFA и HIF1A могут нуждаться в более агрессивной тактике лечения с ранним применением комбинированной терапии

## ЗАКЛЮЧЕНИЕ

Результаты данного исследования подчеркивают значимость изучения генетических факторов агрессивного течения аденом гипофиза. Определение полиморфизмов генов VEGFA, TP53_2, HIF1A и IL-17A может иметь важное диагностическое и прогностическое значение, позволяя более точно стратифицировать пациентов и разрабатывать персонализированные стратегии ведения.

Авторы декларируют отсутствие явных и потенциальных конфликтов интересов, связанных с содержанием настоящей статьи.

## ДОПОЛНИТЕЛЬНАЯ ИНФОРМАЦИЯ

Участие авторов. Все авторы одобрили финальную версию статьи перед публикацией, выразили согласие нести ответственность за все аспекты работы, подразумевающую надлежащее изучение и решение вопросов, связанных с точностью или добросовестностью любой части работы.

Благодарности. Авторы выражают благодарность сотрудникам лаборатории молекулярно-генетического отдела Специализированного научно-практического медицинского центра гематологии МЗ.РУз, в частности профессору Бобоеву А.Т. за содействие в проведении молекулярно-генетических исследования.
